# Long-term survival with pemetrexed-based chemotherapy in a patient with metastatic lung adenocarcinoma of unclear primary origin harboring MTHFR C677T(T/T) mutation: a case report

**DOI:** 10.3389/fonc.2024.1435357

**Published:** 2025-01-21

**Authors:** Yuan Yu, Nan-Jing Li, Jin Wang

**Affiliations:** ^1^ Division of Thoracic Tumor Multimodality Treatment and Department of Medical Oncology, Cancer Center, West China Hospital, Sichuan University, Chengdu, Sichuan, China; ^2^ West China Medical School, Sichuan University, Chengdu, Sichuan, China; ^3^ West China Biomedical Big Data Center, Sichuan University, Chengdu, Sichuan, China; ^4^ Division of Radiotherapy, Cancer Center, West China Hospital, Sichuan University, Chengdu, China

**Keywords:** pemetrexed, next-generation sequencing, adenocarcinoma, lung cancer, MTHFR, polymorphism

## Abstract

This case report presents a patient with metastatic adenocarcinoma of unclear primary focus at initial presentation and revealed lung adenocarcinoma in subsequent follow-up. The patient has been surviving for more than 10 years after pemetrexed-based treatment and local radiotherapy. Sequential gene tests showed kirsten rat sarcoma viral oncogene homolog (KRAS) G13D mutation and epidermal growth factor receptor (EGFR) 19ins. To further investigate the correlation between pemetrexed efficacy and genetic polymorphisms, genotyping tests on folate-metabolism-related genes [methylenetetrahydrofolate reductase (MTHFR) (C677T) and MTHFR (A1298C)] were performed, revealing that the patient exhibited the T/T genotype for MTHFR (C677T) and the A/A genotype for MTHFR (A1298C). The clinical data and gene analysis were discussed with literature review to explain the underlying explanation for the long survival.

## Introduction

Patients with bone metastases from lung adenocarcinoma and who do not have driver gene mutations typically have a median survival period of approximately 6 to 7 months, and only 10% of them survive for 1 year (1, 2); for tumors with unknown primary lesions, the average median survival period is only 6–9 months, and the median survival period during chemotherapy is about 6–14 months (3–5). Previous studies have shown the importance of finding the primary lesion; ^18^F-PET-CT and NGS provide technical support. Pemetrexed is widely used in thoracic tumors (malignant pleural mesothelioma and lung cancer) with a median survival of about 12.6 months for advanced lung adenocarcinoma (6). The present case of long-term survival in the kind of disease is rare. As a multi-targeted antifolate, pemetrexed plays a role in disturbing folic acid metabolism, impacting on DNA methylation, affecting the synthesis of methionine and nucleic acids, and substantially affecting cell repair and proliferation (7). Due to the importance of identifying predictive factors for the clinical outcome of pemetrexed, scientists have conducted multifaceted studies (8, 9). In the meantime, there are other possible pemetrexed drug resistance mechanisms relevant to DNA damage and repair as well as drug dynamics (10). Polymorphisms that play a role in pemetrexed transport and nucleotide metabolism might influence the clinical outcome as well. MTHFR plays a crucial role as an enzyme in the metabolic pathway of folate, and the 677C > T polymorphism of the MTHFR is a significant single-nucleotide variation that has been associated with the response to pemetrexed-based chemotherapy. Feng Han et al., in their meta-analysis, showed that NSCLC patients with TT or CT genotype had a better partial response but had an increased risk for disease progression, which remains controversial (11, 12). There is still a lack of clear evidence to clarify the inconsistent efficacy of pemetrexed due to the complexity of the biology, the multiple drug pathway, and the cofactors of gene–gene interaction. In this report, we outline the diagnosis and treatment procedures while also conducting a literature review to enhance the comprehension of this disease. The aim is to offer clues for its clinical management and further research endeavors.

## Case presentation

### Background

**Figure 1:**
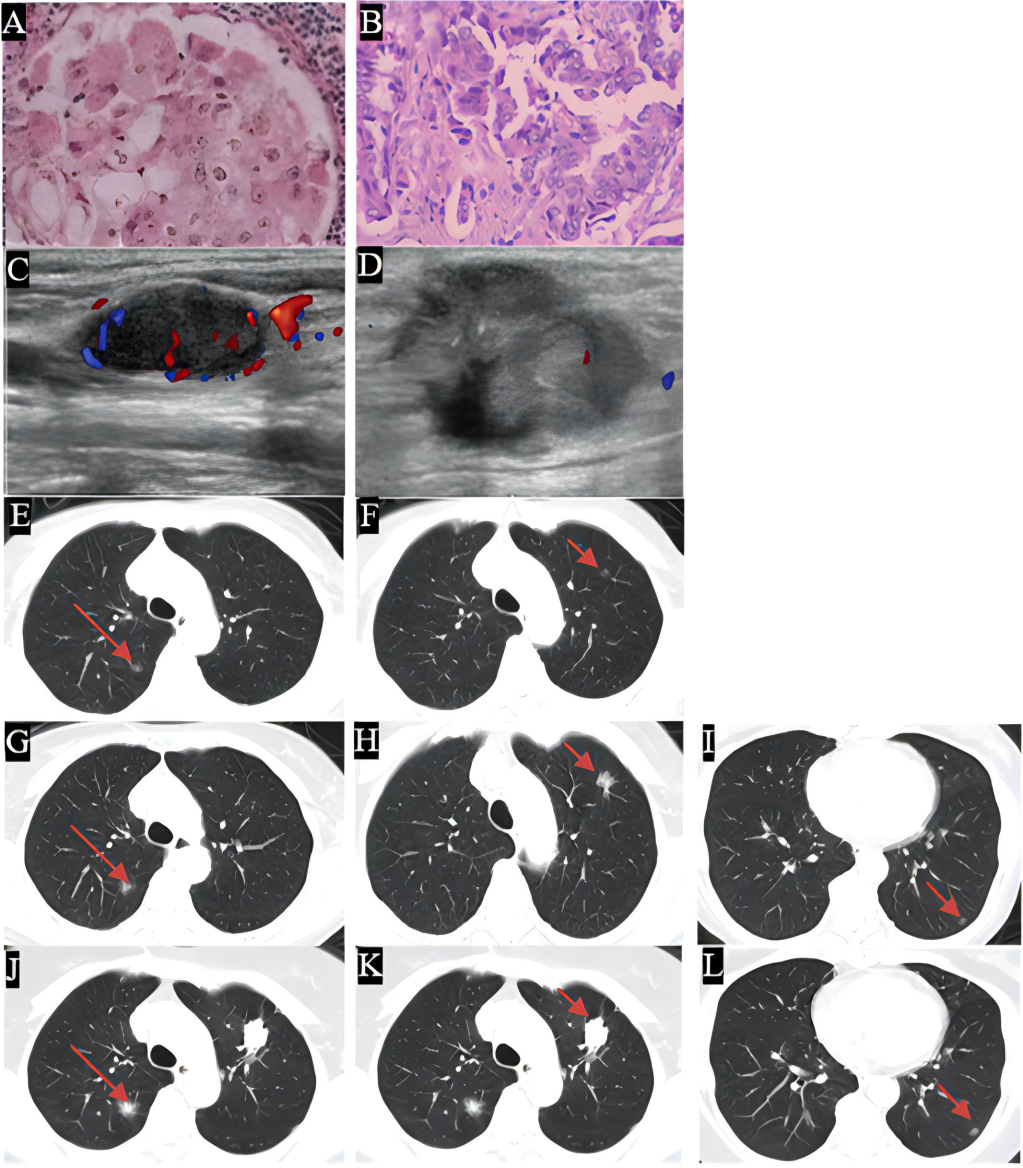
The histological examination of the specimen was depicted, with images **(A, B)** showing the H&E stained sections. Images **(C, D)** displayed the enlarged cervical lymph nodes, in 2019 this patient restarted the pemetrexed based chemotherapy in combination with bevacizumab, followed by radiotherapy for cervical lymph nodes and immunotherapy and achieved a relatively stable therapeutic effect. Images **(E–L)** illustrated the progression of grass ground nodules in the lung from 2013 to 2024. Specifically, images **(E, G, J)** indicated an increase in size of nodule A, while images **(F, H, K)** demonstrated the growth of nodule B, a lung puncture was done in 2024 due to the increasing solid component, thus EGFR19ins mutation was found and the afatinib was given. Images **(I, L)** depicted the presence of nodule C. Over the course of the extended treatment, minor nodules began to appear and gradually increased in size at a slow rate which reflected the complexity of the tumor background.

**Figure 2:**
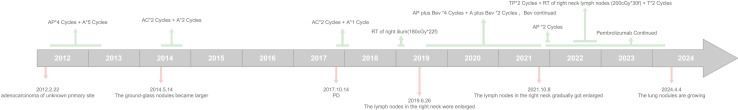
Timeline diagram of the patient's treatment history. (A, Pemetrexed; AP, Pemetrexed+ Cisplatin; AC, Pemetrexed+ Carboplatin; RT, radiothenapy; Bev, Bevacizumab; TP, Albumin-paclitaxel micelles+ Carboplatin; T, Albumin-pacitaxel micelles + Carboplatin).

In February 2012, a 54-year-old Chinese woman was unintentionally found to have enlarged left cervical lymph nodes and diagnosed with metastatic poorly differentiated adenocarcinoma after cervical lymph node dissection biopsy (**Figures 1A, B**). The EGFR gene mutation analysis [Double-Stranded Probe Hybridization (13)], including EGFR 19-Del, L858R, T790M, 20-Ins, G719X, S768I, and L816Q revealed negative results (**Figure 2** illustrates the patient’s treatment history). The patient completed a baseline examination of ^18^F-PET-CT revealing a 9-mm solid nodule in the lower lingual segment of the left upper lung, multimal mediastinal, hilum, and bilateral supraclavicular lymph node combined with the right 5th rib, and bilateral ilium metastases.

### Treatment history

**Figure 3:**
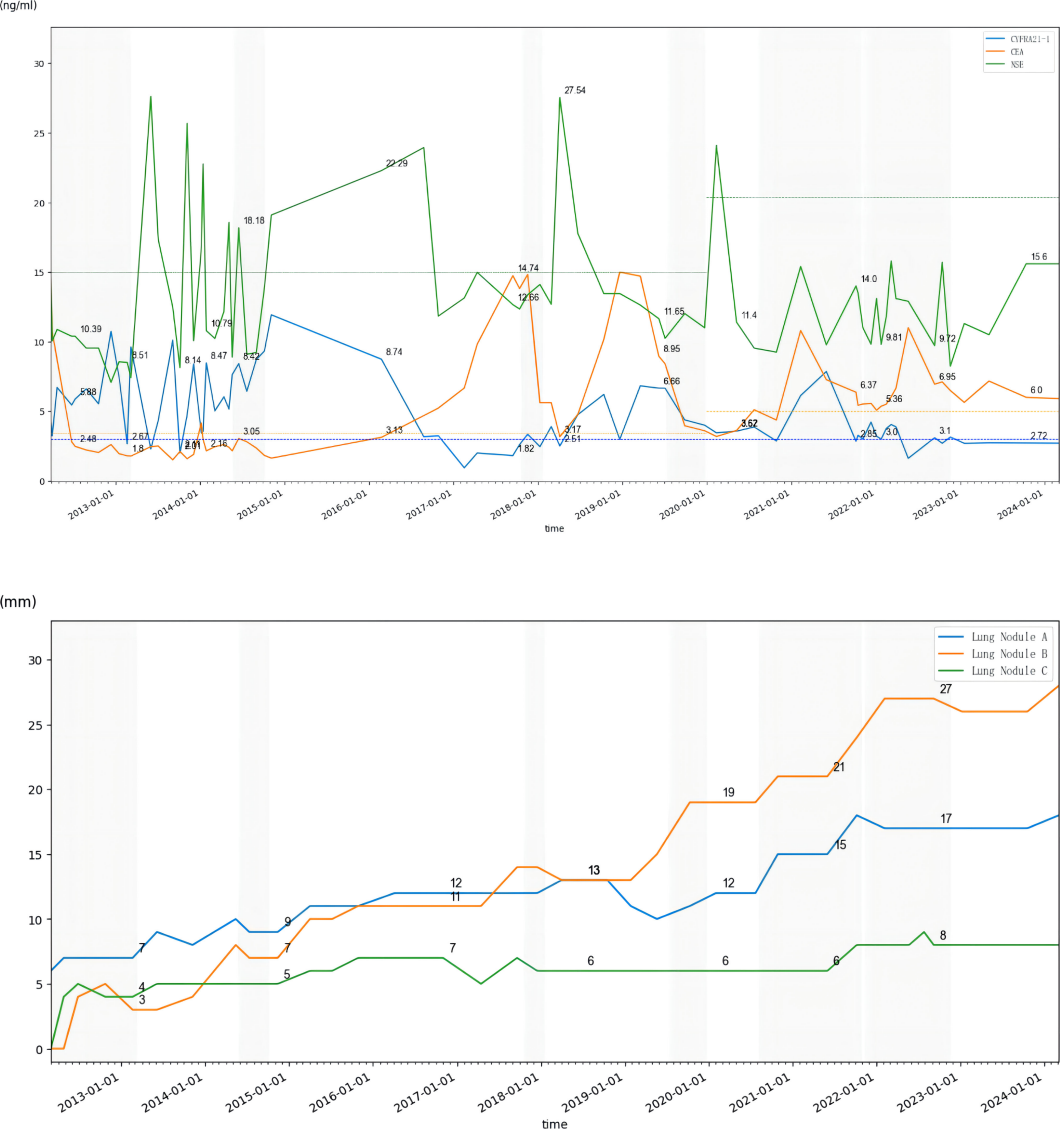
Trends in tumor markers from 2012 to 2024 (dotted line represents upper limit of normal) and diameters of the three largest lung nodules in CT scans from 2012 to 2024 (shading represents the period of receiving chemotherapy); Nodule A: located in the posterior segment of the right upper lobe of the lung; Nodule B: located in the anterior segment of the upper lobe of the left lung; Nodule C: located in the posterior basal segment of the left lower lobe of the lung.

**Figure 4:**
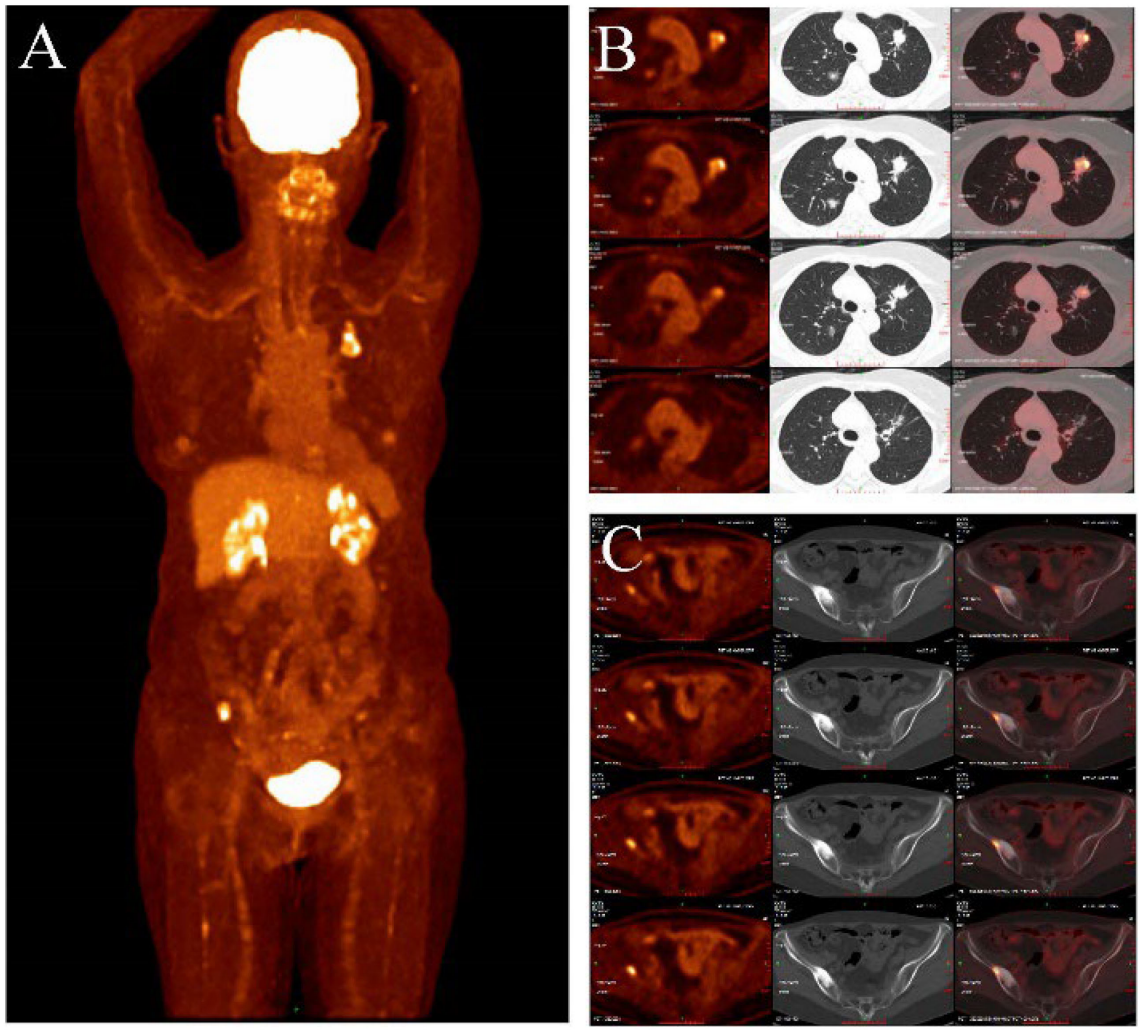
**(A–C)** showed the PET/CT (2024-04-12): In the anterior segment of the left upper lobe of the lung and the posterior segment of the right upper lobe, there are mixed ground-glass nodules, with the largest measuring approximately 22x20mm and 18x13mm, respectively **(B)**. The lesions mentioned above exhibit abnormally increased uptake with a maximum SUV of 7.36. There are scattered small nodules in both lungs with a long diameter ranging from approximately 3-11mm, showing no abnormal increase in F-FDG uptake. No abnormal increase in 18F-FDG uptake is observed in the bilateral breasts, mediastinal lymph nodes, bilateral pleura, or esophagus. The maximum SUV of the mediastinal blood pool is 2.05, with an average SUV of 1.84. CT scan reveals no enlargement of the mediastinal lymph nodes, no thickening of the bilateral pleura, and no pleural effusion in the chest cavity. Increased bone density was observed in the right iliac bone, with a maximum SUV of 8.51 **(C)**.

Pemetrexed (800 mg d1) plus cisplatin (40 mg d1–d3) q3w for 4 cycles and 5 cycles of single-pemetrexed (800 mg d1 q3w) regimen was initiated, and the original nodule in the upper lobe of the original left lung was significantly reduced. However, some small nodules emerged, during which the efficacy was evaluated as SD. **Figure 3** illustrates the changes in blood tumor markers and the three most prominent lung nodules observed in CT scans; it may be a reflection of the prognosis. ^18^F-PET-CT was done again in 2014, showing nodules in both the upper right and the left lung which increased in size. The patient restarted chemotherapy with AC (pemetrexed 740 mg d1+ carboplatin 380 mg d1, q3w) and maintenance A (740 mg d1 q3w) regimen for 4.3 months. During the following regular reexaminations, part of the lesions shrank, while the others were enlarged slightly in size. In October 2017, the patient suffered from disease progression and turned on AC and single-agent A regimen of chemotherapy for 3 months and was evaluated with a stable disease in the following 1.6 years. In 2019, the patient underwent local radiation therapy (180cGy*22f) due to pain of the right side of the ilium. In June 2019, she underwent resection of the enlarged right lower neck lymph node which demonstrated adenocarcinoma from the lung according to immunohistochemistry [PCK (+), CK7 (+), TTF-1 (+), NapsinA (+), CEA (+), E-C (+), CK20 (-), CDX-2 (-), PAX-8 (-), ALK -V (-), and ROS-1 (-)] (**Figures 1C, D**). Peripheral blood 1021 next-generation sequencing (NGS) test (Guangzhou Huayin Healthcare Group Co.) (14) revealed KRASp.G13D mutation (abundance: 0.8%); TMB (intermediate), MSS. The patient was treated for 16 months with pemetrexed (750 mg d1) + cisplatin (40 mg d1–d3) + bevacizumab (400 mg d1) q3w, pemetrexed+ bevacizumab, and single bevacizumab regimen, during which the efficacy was evaluated as SD. Then, due to the gradual enlargement of the lung lesions (**Figures 1E–L**), the patient underwent 2 cycles of pemetrexed + cisplatin + pembrolizumab (200 mg d1) q3w in November 2021. Due to the enlargement of the right cervical lymph node, the treatment was switched to albumin–paclitaxel micelles (300 mg d1) + carboplatin (40 mg d2) q3w and sequential cervical lymph node radiotherapy (200cGy*30f) plus 2 cycles of single albumin–paclitaxel regimen. Moreover, the patient was continuously treated with monthly immunotherapy using pembrolizumab from 2021 until March 2024, during which there was a slight increase of solid component in the lung nodules. Another ^18^F-PET/CT (**Figure 4**) was done, and needle biopsy of the left upper lung nodule showed lung adenocarcinoma. The NGS (the Precision Medicine Center, West China Hospital of Sichuan University) showed mutations of EGFR19ins\MDM2\ATRX, TMB (low), MSS. Thus, the patient was switched to oral afatinib (30 mg daily) treatment.

### Further exploration and patient’s status

To explore the association between prognostic significance and genetic polymorphisms, we performed genotyping tests on folate-metabolism-related genes (MTHFR (C677T) and MTHFR (A1298C)) for the patient. The results showed that the patient exhibited the T/T genotype for MTHFR (C677T) and the A/A genotype for MTHFR (A1298C). The patient’s treatment toxicities were mainly vomiting and mild hypertension (recovered immediately after finishing bevacizumab) and grade 1 myelosuppression (no medication required) during the whole treatments. She continued receiving treatment with an ECOG PS score of 0–1 and an NRS score of 0.

## Discussion

In this case, the patient presented with metastatic poorly differentiated adenocarcinoma of the neck which did not indicate any primary tumor site. Studies have shown lung adenocarcinoma to be the most common type of CUP, and occult primary lung tumors are found in approximately 17%–27% of early autopsy cases (15). PET/CT acts a role in comprehensively detecting tumor biological activity, guiding tissue biopsy sites, reducing invasive procedures, evaluating oligometastatic foci, and orienting treatment in patients with CUP (16). Previous studies have shown that ^18^F-PET/CT detected about 33% of primary tumor sites, among which the most common primary site remains the lung, followed by nasopharynx and pancreas (17). Technological innovation brought breakthrough—for example, new agent ^68^Ga-FAPI-PET/CT can detect lesions that cannot be detected by ^18^F-PET/CT, especially in the head and neck region (18, 19).

Bone, one of the most common metastatic sites, accounts for the presence of bone metastases in 70% of advanced breast cancer cases, and more than 40% of advanced lung and urological tumors combined with bone metastases. The treatment of lung cancer (no driver gene mutation) combined with bone metastasis is mainly palliative and has a low survival rate, with a median survival of about 6 to 7 months after detection, with one-year survival rate of approximately 10% (1, 2). However, in this case, the tumor seemed to enter a “dormant phase” after chemotherapy, and survival of more than 10 years was surprisingly achieved. Maybe we can find some clues with the help of molecular sequencing, which may help to identify the possible underlying clues.

Prediction of CUP by molecular sequencing has been shown in some studies, with lung cancer-related mutations detected in about 11%–12% of patients (20, 21). Approximately 30%–85% of CUP patients harboring relevant genetic mutations (22, 23), among which TP53, KRAS, and CDKN2A are the most common (24). A prospective single-arm phase II clinical study showed that a specific treatment via NGS may improve survival, with a 1-year survival rate of 53.1%, a median OS of 13.7 months, and a median PFS of 5.2 months (25). Meanwhile, there are no prospective randomized clinical trials demonstrating whether specific or non-specific treatments are more efficacious. This patient underwent three sequential gene tests throughout the extended clinical course, one initial tissue PCR aiming only seven EGFR mutations, one later blood NGS of 1,021 genes which found a rare KRAS p.G13D mutation, and one latest tissue 1,021 genes NGS of lung nodule needle biopsy which illustrated EGFR 19ins. Studies have shown that the concordance of tissue and blood NGS of primary tumor metastatic lymph node in primary treated metastatic NSCLC is about 62%–78% (26–28). It has been shown that blood NGS is more likely to detect ctDNA in patients with a higher level of tumor markers and is more likely to find out the heterogeneity missed by tissue biopsy (29–31). The tumor markers of this patient were relatively low during the course of the disease, which matched the slow progression; however, that might lead to false negative or false positive gene detection in the meantime (**Figure 3**).

KRAS G13D, as one of the KRAS gene isoforms, accounts for a relatively small number of LUAD (3%) (32). Most of the patients with combined KRAS gene mutations have a poor prognosis. Recently, basic experiments have developed biochemically selective KRAS G13D inhibitors, laying the foundation for new drug discovery and development (33).

KRAS G13D is a biomarker of sensitivity to EGFR-TKIs due to its lack of affinity for NF1 (32). EGFR exon19 insertions which can lead to IL3-independent cell growth were commonly detected in lung cancer. Research showed that it may be sensitive to EGFR TKIs such as gefitinib, afatinib, osimertinib, etc. (34–36). Advancements in genetic testing technology and its increased availability have enabled the detection of co-mutations of EGFR and KRAS in some patients. The presence of KRAS mutations alongside other critical gene alterations illuminates the potential for synergistic combination therapies as well as targeting downstream effectors such as MEK and PI3K (37, 38).

**Supplementary Table S1** shows the results of tissue NGS in 2024. There was a huge difference between the previous results of blood NGS (**Supplementary Table S2** shows a list of the mutations without relevant medication information or of undermined clinical significance in 2019).

As a part of downstream signal, the acquired mutation of KRAS in metastatic lung cancer can contribute to the resistance to EGFR-TKIs, affecting survival (39, 40). On the other hand, the pivotal function of the KRAS G12V mutation in mechanisms of resistance to osimertinib has been confirmed through a cellular test (41).

The efficacy of anti-PD1 inhibitor for patients harboring KRAS has been reported to correlate with the combined mutating genes (42). Patients with rare KRAS mutations have worse PFS and OS than those with common KRAS mutations when immunotherapy was not used (with a median PFS of 3.4 months and median OS of 5.2 months), and a high PD-L1 expression can elevate the efficacy of ICIs in these patients (43). In addition, this patient received concurrent regional radiotherapy which may enhance the efficacy of pembrolizumab. A combined analysis of two randomized trials has revealed that the incorporation of radiotherapy to pembrolizumab-based immunotherapy notably enhanced both response rates and clinical outcomes for individuals with metastatic NSCLC. Specifically, the median PFS improved from 4.4 to 9.0 months (*p* = 0.045), and the median OS extended from 8.7 to 19.2 months (*p* = 0.0004) (44, 45). It is interesting to note that this patient got a relatively high level of MDM2 amplification which codes for an E3 ubiquitin ligase that is localized in the nucleus. This protein could facilitate the development of tumors by directing tumor suppressor proteins, such as p53 (46, 47). Kato et al. found that of 155 cancer patients on immunotherapy, the six who failed treatment within 2 months (all on PD-1/PD-L1 inhibitors) had MDM2/MDM4 amplification. Four patients’ tumors grew by 55% to 258% in that time, suggesting that MDM2 amplification might initiate signal cascades causing hyperprogression (47). The study of APG-115 in combination with pembrolizumab shows the potential of combining MDM2-P53 inhibitors with immuno-oncology drugs. In 26 patients, two had complete responses and four had partial responses, giving an overall response rate of 23.1% (48). However, our case had no hyperprogression associated with MDM2 amplification.

In our case, the patient has a surprisingly long survival of nearly 10 years with pemetrexed-based chemotherapy. To optimize the treatment strategy, a comprehensive understanding and empirical evidence of the drugs are essential. Pemetrexed is a multi-targeted antifolate drug, which plays an anti-tumor role by interfering with cellular folate metabolism and inhibiting nucleotide synthesis, and its multi-targeted characteristic may contribute to the broad spectrum of anti-tumor activity (49). Pemetrexed combined with cisplatin is one of the commonly used standard chemotherapy regimens for advanced NSCLC, and phase III clinical trials compared the efficacy and toxicities of the regimen with gemcitabine combined with cisplatin in patients with metastatic chemotherapy-naive NSCLC. Adenocarcinoma patients had a greater survival benefit with pemetrexed, with a median survival of 12.6 months, and it also possesses lower toxicity and is highly convenient. Phase II clinical trials and retrospective studies in Japan showed that the response rate of this regimen was about 44.0% and 37.5%, with median PFS of 4.3 and 5.6 months and median OS of 22.2 and 18.8 months, respectively (6, 50–52). Maintenance therapy with pemetrexed in patients with advanced NSCLC after four to six cycles of first-line chemotherapy has also been advised, with meta-analysis suggesting that pemetrexed significantly prolonged PFS (HR = 0.54, *p* = 0.000) and OS (HR = 0.75, *p* = 0.000) compared with BSC (53, 54). In the ECOG-ACRIN 5508 study, the median OS of bevacizumab maintenance was 14.4 months, with 16.4 months of bevacizumab plus pemetrexed, which matched in this case (55).

To understand the possible mechanism underlying the efficacy of pemetrexed, prior studies have identified several indicators that suggest the effectiveness of pemetrexed across different aspects. Retrospective research showed that in M1a stage cancer, reduced thymidylate synthase levels, lower levels of CEA at baseline, and the return of CEA levels to normal after treatment with Pem-Cis (pemetrexed plus cisplatin) were indicators of a sustained positive response to pemetrexed maintenance therapy (56). The lower CEA level may possibly account for the better efficacy in our case. Some case reports demonstrated the success of pemetrexed in combination with platinum agents in some complicated cases (57). Although studies predicted drug resistance from aspects of oncogene mutation, DNA synthesis, and replication damage and repair, as well as drug dynamics (10), there are no reliable biomarkers to predict the efficacy of this regimen (58). Basic experiments show that upregulation of hsa-miR-320a-3p may be associated with the anti-tumor effects of pemetrexed, suggesting drug efficacy relevance at a non-genetic level (59). Previous clinical studies have shown that pemetrexed-containing chemotherapy regimens in NSCLC have comparable efficacy for chemotherapy in patients with mutations in RET, ALK, ROS1 genes, and TTF-1 positive status (60, 61).

Thymidylate synthase (TS), dihydrofolate reductase (DHFR), and glycinamide ribonucleotide formyl transferase (GARFT), along with ribonucleotide reductase M1 (RRM1) and methylenetetrahydrofolate reductase (MTHFR), are primary targets of pemetrexed, which play roles in DNA synthesis and replication. Their elevated levels indicated a weaker response to pemetrexed. In this present study, this female adenocarcinoma patient exhibited the T/T genotype for MTHFR (C677T) and the A/A genotype for MTHFR (A1298C) survived for over 12 years. It is worthy to identify the mechanism that promoted longer survival in specific patients. The association between clinical outcomes and genetic polymorphisms is inconclusive, owing to factors such as the limited sample size, ethnic diversity, interactions between genes, and the mutual effects metabolism pathways; target gene mutations are not fully detected (62–64). Weiwei Tong et al. did research for female Chinese population in one hospital of North China and found that the TT genotype of C677T and AA genotype of A1298C increased the risk of NSCLC (65). R. Zhong et al. did a meta-analysis showing that North Chinese populations with MTHFR C677T polymorphism tend to be more susceptible to lung cancer (66). N. Zhu et al. did a meta-analysis and said that male TT homozygote carriers showed increased susceptibility, while the allelic contrast and homozygote model had a protective effect in females (67). A meta-analysis revealed that the MTHFR C677T polymorphism was significantly associated with an increased risk of lung cancer in both Asian and global populations, yet this association was not observed in Caucasian populations—TT vs. CC, OR: 1.518, 95%CI = 1.220–1.890; CT vs. CC, OR: 1.053, 95%CI = 0.940–1.179 (68). Gaochen Lan conducted a retrospective research in 51 patients with advanced non-squamous NSCLC and stated that the MTHFR C677T polymorphism may be considered a predictive factor for these specific toxicities—leukopenia, neutropenia, nausea, and fatigue in NSCLC patients undergoing treatment with single-agent pemetrexed (PEM) (69) while Yu Bai et al. did a meta-analysis showing that the MTHFR 677 C > T polymorphism did not serve as a predictive marker for the efficacy of pemetrexed (PEM) in non-small cell lung cancer (NSCLC) patients but that the T allele may increase the risk of hematological toxicity (11). One retrospective analysis presented five polymorphisms in TS, MTHFR, and ERCC1 genes as molecular predictive markers for non-squamous NSCLC patients treated with pemetrexed, and platinum front-line chemotherapy showed the integrated analysis of the TS gene’s VNTR polymorphism and the MTHFR 677C>T indicated that patients who concurrently possessed the 3R allele in the TS gene and two C alleles in the MTHFR gene experienced a reduction in PFS (70). Thus, it is worthy to see more large-scale clinical trials in the future to discover its mechanism.

It is worthwhile discussing the patient’s performance status and its prognostic significance. Despite the patient’s initial diagnosis of bone metastases in the rib and bilateral iliac, it was the enlarged cervical nodule that served as a reminder of her condition. Throughout the extensive and sometimes rigorous treatment regimen, she maintained a relatively good performance status score of 0–1. Studies had demonstrated that PS is an independent prognostic parameter (71). A randomized phase III trial by Kosmidis et al. revealed that the median survival of patients with a PS of 2 was significantly lower compared to those with a PS of 0–1 (5.9 vs. 11.1 months, *P* < 0.0001) (72). Furthermore, an ECOG phase III trial indicated that patients with a PS of 2 experienced a higher incidence of adverse events and significantly poorer survival, with a median survival rarely exceeding 5 months, compared to those with a PS of 0 or 1, and with 1-year survival rates below 20% (73). A meta-analysis by Bartłomiej Tomasik showed that advanced NSCLC patients with impaired performance status were, on average, twice as unlikely to respond to immune checkpoint inhibitors (ICIs) as those with a representative PS of ≤1 (74). In this case, the PS 0–1 score reflected better treatment efficacy and lower adverse event rates and contributed to long-term survival.

## Conclusion

The patient in this case was assumed to primarily have a poor prognosis indicated by the bone and multiple lymph node metastases at baseline while achieving a long-term survival with pemetrexed-based chemotherapy, with mild side effects. Although the underlying mechanism is unclear, a thorough grasp of medical history, disease trajectory, and quality of life is essential for effective disease management throughout the entire course. Additionally, employing machine learning to forecast outcomes may enhance the predictive capabilities of this comprehensive approach (75, 76). The inconsistency of the tumors’ response in various sites according to the patient’s work-up reflects the high degree of heterogeneity. The mutations in the KRAS gene may be a factor contributing to drug resistance and progression. On the other hand, it may be related to the efficacy of pemetrexed. The result of NGS may be the clue. Proper time of NGS can facilitate more informed clinical decisions and underlying causes and reflect the progression of the disease, particularly in the absence of clear markers. Multi-disciplinary modality is essential for the control of CUP.

## Data Availability

The original contributions presented in the study are included in the article/**Supplementary Material**. Further inquiries can be directed to the corresponding author.

## Glossary

CUP: cancer of unknown primary
PET/CT: positron emission tomography–computed tomography
TMB: tumor mutational burden
MTHFR: methylenetetrahydrofolate reductase
SNPs: single-nucleotide polymorphisms
MSS: microsatellite stability
NGS: next-generation sequencing
NSCLC: non-small-cell lung cancer
HLA: human leukocyte antigen
EGFR: epidermal growth factor receptor
SD: stable disease
PD: progressive disease
ORR: objective response rate
AP: pemetrexed plus cisplatin
single-A regimen: pemetrexed
AC: pemetrexed plus carboplatin
T: micelles of albumin-bound paclitaxel
ECOG PS: Eastern Cooperative Oncology Group Performance Status
NRS: numeric rating scale
TTF-1: thyroid transcription factor-1
PFS: progression-free survival
OS: overall survival
TS: thymidylate synthase
DHFR: dihydrofolate reductase
GARFT: glycinamide ribonucleotide formyl transferase
RRM1: ribonucleotide reductase M1
MTHFR: methylenetetrahydrofolate reductase
KRAS: kirsten rat sarcoma viral oncogene homolog
ALK: anaplastic lymphoma kinase
ROS1: ROS proto-oncogene 1
RET: rearranged during transfection
H&E: hematoxylin and eosin
FR-α: folate receptor alpha
HR: hazard ratio
BSC: basic supportive care
FPGS: folylpolyglutamate synthase gene
PD-L1: programmed cell death 1 ligand 1
LUAD: lung adenocarcinoma
CRC: colorectal cancer
PDAC: pancreatic ductal adenocarcinoma
