# Supplementary material for: Long-term survival with pemetrexed-based chemotherapy in a patient with metastatic lung adenocarcinoma of unclear primary origin harboring MTHFR C677T(T/T) mutation: a case report
Source: Front Oncol. 2025 Jan 21;14:1435357. doi: 10.3389/fonc.2024.1435357 (PMC11790433; doi:10.3389/fonc.2024.1435357)
Supplement: Supplementary file 1 [file Table1.docx]

| gene | type | place | frequency |
| --- | --- | --- | --- |
| ATRX | nonsense mutation | NM_000489.3: exon 18:c.4924C>T(p.Q164  2*) | 7.8% |
| EGFR | Insertion mutation | NM_005228.3: exon 19:c.2219_2236dupTT  CCCGTCGCTATCAAGG (p.K745_E746insVPVAI) | 7.5% |
| MDM2 | gene amplification | NM_002392.4 | 8.2% |
| FAM123B | missense mutation | NM_152424.3:exon 2:c.2860G>T (p.G954W) | 2.0% |
| FANCM | missense mutation | NM_020937.2:exon 5:c.968G>C (p.R323T) | 1.8% |
| FYN | missense mutation | NM_153047.3:exon 7:c.1000C>T (p.P334S) | 1.7% |
| FAT1 | missense mutation | NM_005245.3:exon 2:c.2902G>T (p.D968Y) | 1.5% |
| ERBB4 | missense mutation | NM_005235.2:exon 27:c.3420C>A (p.S1140R) | 1.4% |
| PIK3CG | missense mutation | NM_002649.2:exon 2:c.1951T>A (p.L651M) | 1.2% |
| BRAF | missense mutation | NM_004333.4:exon 16:c.1929_1930delAAinsGT (p.I644F) | 1.1% |
| RET | missense mutation | NM_020975.4:exon 3:c.397C>T (p.R133C) | 1.1% |
| PRF1 | missense mutation | NM_001083116.1:exon 3:c.778G>T (p.V260F) | 1.0% |
| IFNG | gene amplification | NM_000619.2 | 6.0 copies |
| BTK | gene amplification | NM_000061.2 | 5.4 copies |

**Table.1** Organic NGS results of pulmonary puncture in 2024.

| gene | type | place | frequency |
| --- | --- | --- | --- |
| ASXL1 | nonsense mutation | NM 015338.5:exon 7:c.664G>T(p.E222*) | 9.4% |
| KDM6A | missense mutation | NM 001291415.1:exon 18:c.2242C>G(p.L748V) | 6.3% |
| SETD2 | missense mutation | NM 014159.6:exon 3:c.2428G>C(p.E810Q) | 5.6% |
| SPEN | missense mutation | NM 015001.2:exon 11:c.6433G>C(p.D2145H) | 6.1% |
| SPEN | missense mutation | NM 015001.2:exon 11:c.7164G>C(p.E2388D) | 7.9% |
| SPEN | Frameshift deletion | NM 015001.2:exon 11:c.7225delG(p.2409fs) | 7.4% |
| SPEN | missense mutation | NM 015001.2:exon 11:c.8434G>A(p.E2812K) | 7.7% |
| TMPRSS2 | nonsense mutation | NM 001135099.1:exon 6:c.586C>T(p.Q196*) | 5.5% |
| MCL1 | gene amplification | 1q21.3 | 4.8copies |
| SDHA | gene amplification | 5p15.33 | 7.6copies |

**Table.2** Mutations without relevant medication information or of undetermined clinical significance of blood NGS in 2019.
